# Three Cases of Electrical Traumatic Neurosis1Read in Buffalo before the Medical Association of Central New York.

**Published:** 1891-10

**Authors:** James W. Putnam

**Affiliations:** Professor of Nervous Diseases, University of Buffalo; 388 Franklin Street


### THREE CASES OF ELECTRICAL TRAUMATIC NEUROSIS.[1]

By JAMES W. PUTNAM,
Professor of Nervous Diseases, University of Buffalo.

In July, 1890, during a severe thunder and lightning storm, three telephone operators received an electric shock, from an unusually vivid flash that passed through the lightning cut-off with which the key-board is supplied. At the time of the shock each operator was wearing a metallic arrangement closely fitted to the head, which holds the receiving phone in position against the ear. They were each holding a metal plug in the hand, preparing to make a connection on the key-board.

No. 1 was a large, healthy, well-developed young woman of Irish descent, aged 20. She was rendered unconscious for a few moments. On regaining consciousness, she complained of severe headache, and of deafness of the left ear, which was in contact with

1. Read in Buffalo before the Medical Association of Central New York.

the phone, and which received the shock. Examining this patient, I found complete cutaneous anesthesia of the left side, the pharynx and conjunctivæ. There was no paralysis. From the symptoms I diagnosticated the deafness to be functional, and treated it as such by suggestion, with the result that the hearing was perfectly restored. The headache remained, and only yielded to bromide of potassium.

No. 2. A young woman of German parentage, with previous good health. She was in bed when I first visited her. She said it made her dizzy to stand up, and made her headache worse. Examination showed cutaneous anesthesia of left side of face and scalp, left side of thorax anteriorally, left arm, conjunctivæ anesthetic. The anesthesia disappeared after two treatments with the faradic brush, briskly applied. In addition she took a spinal douche, 40 degrees, 15 seconds, daily. The headache was treated with 20 grs. pot. brom. and 15 drops ergot. The headache disappeared, but always came back if she attempted work at the telephone office. Believing this to be a headache suggested to her by the sight of the apparatus from which she received the shock, I hypnotized her, and suggested that she should have no more headache, either at home or at the telephone office. She has remained well, has returned to work, and has had no return of headache.

No. 3. A slight girl, aged 21, of American parentage; was rendered unconscious for a few moments by the shock, was taken home, and was delirious for one hour, after which she became quiet; complained simply of throbbing headache. Examination discovered anesthesia, as in the others, of the left side. Sensation was restored by one application of the faradic brush; headache disappeared after a few doses of pot. brom.

The similarity of the cases at once strikes us. There was headache and anesthesia in them all, and each showed susceptibility to suggestion. I present these cases, believing them to be a combination of hysterical symptoms of anesthesia, with the headache we naturally expect to result from severe electric shock.

388 Franklin Street.

---

Fermentation of milk occurs only in consequence of the introduction into it of microörganisms. If the milk be received by a sterilized tube into a sterilized receptacle directly from the udder of the cow, it will not ferment or become acid, though kept indefinitely.—*Ex. in Southern Medical Record.*

